# Overview on the Evaluation of the Elastic Properties of Non-Carbon Nanotubes by Theoretical Approaches

**DOI:** 10.3390/ma15093325

**Published:** 2022-05-05

**Authors:** Jorge M. Antunes, André F. G. Pereira, Nataliya A. Sakharova

**Affiliations:** 1 Centre for Mechanical Engineering, Materials and Processes (CEMMPRE), Department of Mechanical Engineering, University of Coimbra, Rua Luís Reis Santos, Pinhal de Marrocos, 3030-788 Coimbra, Portugal; andre.pereira@uc.pt (A.F.G.P.); nataliya.sakharova@dem.uc.pt (N.A.S.); 2 Abrantes High School of Technology, Polytechnic Institute of Tomar, Quinta do Contador, Estrada da Serra, 2300-313 Tomar, Portugal

**Keywords:** non-carbon nanotubes, graphene-like hexagonal lattice, elastic properties, modelling, numerical simulation

## Abstract

Low-dimensional structures, such as nanotubes, have been the focus of research interest for approximately three decades due to their potential for use in numerous applications in engineering and technology. In addition to extensive investigation of carbon nanotubes, those composed of elements other than carbon, the so-called non-carbon nanotubes, have also begun to be studied, since they can be more suitable for electronic and optical nano-devices than their carbon counterparts. As in the case of carbon nanotubes, theoretical (numerical and analytical) approaches have been established predominantly to study non-carbon nanotubes. So far, most of work has dealt with the investigation of the structural and electrical properties of non-carbon nanotubes, paying less attention to the evaluation of their mechanical properties. As the understanding of the mechanical behaviour of the constituents is fundamental to ensure the effective performance of nanotube-based devices, this overview aims to analyse and systematize the literature results on the elastic properties of inorganic non-carbon nanotubes.

## 1. Introduction

The discovery of carbon nanotubes (CNTs) gave rise to studies of the prediction and synthesis of new graphene-like structures based on other elements and chemical compounds, such as nitrides, phosphides and carbides, among others. Compounds of elements of the 12th–15th groups of the periodic table are able to establish a honeycomb diatomic arrangement, forming nanotubes (NTs) with a graphene-like hexagonal lattice. Boron nitride (BN), aluminum nitride (AlN), gallium nitride (GaN), indium nitride (InN), boron phosphide (BP), aluminum phosphide (AlP), gallium phosphide (GaP), indium phosphide (InP) and silicon carbide (SiC) nanotubes are examples of nano-tubular structures beyond the CNTs. These non-carbon nanotubes (N-CNTs), being wide band gap semiconductors or dielectrics, have promising applications in nano-devices for optoelectronics and electronics, such as light emitting diodes (LEDs) [1,2,3], field effect transistors [4,5], sensors and bio-detectors [6,7,8], high-frequency transistors [9] and tips in scanning probe microscopy [10,11]. A number of these non-carbon NTs have already been successfully synthesized, as in the cases of BNNTs [12], AlNNTs [13,14,15], GaNNTs [16,17], GaPNTs [18], InPNTs [19] and SiCNTs [20]; others were predicted theoretically, such as InNNTs [1,21], BPNTs [22,23], and AlPNTs [24].

Until now, work on N-CNTs has been mainly devoted to the study of their stability, structural and electronic properties (see, for example [1,22,23,24,25,26,27,28,29,30]). Regarding the investigation of their mechanical behaviour, studies are at a relatively early stage, due to the complexity of accurately measuring the mechanical properties, whose study needs to be in-depth and diversified. With the exception of certain works dealing with the evaluation of the mechanical properties of boron nitride NTs (see, for example [31,32,33,34,35,36]), the other inorganic NTs have received noticeably less research attention [29,37,38,39,40,41,42,43]. This lack of knowledge about the mechanical behaviour of N-CNTs is associated with difficulties in building robust and effective nanotube-based devices.

Since the experimental procedures for materials characterization at the nanoscale are costly and resource-intensive, the investigation of the mechanical behaviour of non-carbon nanotubes is carried out in a more theoretical way, with recourse to analytical and numerical techniques, as in the case of carbon nanotubes. Similar to studies on CNTs, there are three classes of theoretical methodologies that are used for the mechanical characterization of N-CNTs, namely: the atomistic approach, comprising, ab initio, molecular dynamics (MD) and tight-binding molecular dynamics (TBMD); the continuum mechanics (CM) approach; and the nanoscale continuum modelling (NCM) or molecular structural mechanics (MSM) approach. A comprehensive review of theoretical approaches for modelling and characterizing the mechanical behaviour of CNTs can be found in the literature [44,45].

With regard to atomistic approaches, ab initio simulation was used by Kochaev [37] to assess the surface Young’s modulus (product of Young’s modulus by the nanotube wall thickness) and Poisson’s ratio of BNNTs, AlNNTs, GaNNTs, AlPNTs and GaPNTs. Baumeier et al. [46] applied ab initio density functional theory (DFT) within self-interaction-corrected (SIC) pseudopotentials to calculate the surface Young’s modulus of BNNTs and SiCNTs. Hao et al. [47] employed ab initio DFT calculations coupled with linear combination of atomic orbitals (LCAO) to study size-dependent mechanical behaviour of the AlNNTs and to calculate their Young’s modulus. The existing MD simulations, performed to evaluate the mechanical properties of N-CNTs, used analytical or empirical potential functions to describe the interactions between atoms, which form a diatomic graphene-like hexagonal lattice. The Tersoff–type potential was used by Kang and Hwang [40] in their MD simulation study to describe the mechanical behaviour of BNNTs, AlNNTs and GaNNTs under compressive loading and to assess their Young’s modulus. Chandra et al. [48], using the Tersoff–type potential, also analysed the thermal vibrational characteristics of BNNTs. In two of their works Jeng et al. [38,49] adopted MD simulation with Tersoff many-body potential to evaluate the mechanical properties of GaNNTs under tension [38,49] and fatigue [38] loadings. Xiong and Tian [50] carried out a comprehensive study on the torsional properties of BNNTs, using MD simulation with Tersoff potential and, compared the force approach and the energy approach for calculation of BNNTs’ shear modulus. Moon et al. [51], Setoodeh et al. [52], Pan and Si [53] and Zhou et al. [54], using MD simulations based on Tersoff potentials, studied mechanical properties, mechanical behaviour under axial compression, tensile behaviour, and elastic and melting properties of the SiCNTs, respectively. Choyal et al. [31], Verma et al. [55], Tao et al. [56] and Ansary and Ajori [57] used MD with Tersoff–Brenner (TB) potential to describe the interactions between boron (B) and nitride (N) atoms, to clarify the effect of the aspect ratio on the Young’s modulus, to evaluate the Young’s and shear moduli and the Poisson’s ratio, to calculate the Young’s modulus, and to study vibrational behaviour of BNNTs, respectively. Wang et al. [58] employed a MD approach, describing the interactions between gallium (Ga) and nitride (N) atoms by Stilliger-Weber potential, to model the mechanical behaviour of GaNNTs under combined tension and torsion and study their failure. Vijayaraghavan and Zhang [35] used an empirical reactive bond order (REBO) to describe the atomic interactions in the BNNTs and studied their mechanical behaviour under tensile loading. Santosh et al. [59] adopted a force—constant approach for describing the B—N interactions under axial compression in the MD simulation study to calculate the Young’s and shear moduli of the BNNTs. Le [43], in MD simulation with harmonic force fields, obtained explicit expressions for the Young’s modulus of the BNNTs and SiCNTs. Regarding other atomistic approaches, Hernandez et al. [60] calculated the Young’s modulus and Poisson’s ratio of BNNTs using TBMD, while Pinhal et al. [29] coupled periodic DFT calculations with the functional B3LYP (Becke, 3-parameter, Lee–Yang–Parr) to assess the elastic constants of AlNNTs and GaNNTs. Zhang et al. [61] used the density-functional-based tight-binding (DFTB) model in combination with MD to evaluate the Young’s and shear moduli of BNNTs.

Works employing the CM approach, which consider the nanotube as a continuum structure, are relatively scarce in the literature to our knowledge. Oh [62] calculated the Young’s modulus and Poisson’s ratio of BNNTs, using a continuum lattice (CL) analytical thermodynamic approach, together with the TB potential. Panchal et al. [63] modelled the single-walled BNNT as a thin walled tube with thickness and investigated the vibrational response of the BNNTs with attached mass at the free nanotube end. Song et al. [64] developed a finite-deformation shell model to study the mechanical behaviour of BNNTs under tension, compression and torsional loads.

The NCM/MSM approach explores the link between molecular arrangement in nanotubes and solid mechanics and considers the bonds between two atoms in the diatomic structure as elements (for example, beams or springs) well described by elasticity theory. With regard to boron nitride NTs, which have received the most research attention up to now, Li and Chou [34], Salavati et al. [65], Ansari et al. [66], Sakharova et al. [36] and Panchal et al. [67] used the beam element to replace the B-N bond within the framework of the NCM/MSM approach, to study the elastic and dynamic properties, elastic moduli and Poisson’s ratio, electromechanical properties, size-dependent elastic properties and vibrational properties of BNNTs, respectively. In a recent study, Zakaria [68] modelled bonds between B and N atoms by two-sectioned beam elements to evaluate the elastic and vibrational properties of the BNNTs. Giannopoulos et al. [69], instead of the beam elements, used spring-like elements in the modelling of the B-N bond to investigate vibrational behaviour of BNNTs. Yan and Liew [70] considered a representative cell assembled by a boron atom connected to three neighbouring nitride atoms by B-N covalent bonds, to model the BNNTs under NCM/MSM approach. Yan et al. [71] assessed fundamental frequencies and elastic moduli by modelling longitudinal and torsional free vibrations of BNNTs, based on the NCM/MSM approach combined with an Euler beam model. Moreover, there are studies using the NCM/MSM approach, which deal not only with BNNTs, but also with other N-CNTs. Genoese et al. [41] combined the NCM/MSM and CM approaches to perform a numerical simulation study of the mechanical behaviour of BNNTs and SiCNTs under tensile, bending and torsional tests, using a “stick-and-spring” model involving Morse and cosine potential functions. In their work Genoese et al. [41] evaluate the Young’s and shear moduli, and Poisson’s ratio of BN and SiC nanotubes adopting a linkage between the “stick-and-spring” and continuum thin shell Donnell models. Jiang and Guo [39] proposed closed-formed analytical solutions based on the “stick-and-spring” model, to study the buckling and assess the surface Young’s modulus and Poisson’s ratio of BN, AlN, GaN, BP, GaP, InP and SiC nanotubes.

The important issue in modelling the N-CNTs’ mechanical behaviour under the NCM/MSM approach is to properly select the force field constants, regarding bond stretching, k_r_, bond bending, k_θ_, and torsional resistance, k_τ_, which are necessary for calculating the elastic properties of elements representing bonds between two atoms in the hexagonal diatomic structure. In the case of the N-CNTs, the computation of the k_r_, k_θ_ and k_τ_ force constants, used as input for theoretical (numerical or analytical) models, is questionable and not as explicit as for CNTs. In the work by Sakharova et al. [36], the influence of input parameters, obtained based on force field constants assessed by different calculation methods, on the results of finite element (FE) modelling of the mechanical behaviour of BNNTs was studied. A significant scattering of elastic properties was reported. Among the most well-established and commonly used methods for calculation of the force field constants of N-CNTs are those based on UFF (Universal Force Fields) [72], DREIDING force field [73] and ab initio DFT computations, combined with the analytical expressions resulting from molecular mechanics (MM) models [32,33,41]. Jiang and Guo [39] used this methodology, based on the combination of ab initio DFT computations and MM models, to calculate the out-of-plane torsion force constant, which is a component of the torsional resistance force constant, for a wide class of N-CNTs, including BN, AlN, GaN, BP, GaP, InP and SiCNTs.

Although almost all the developments in the mechanical characterization of N-CNTs have been achieved with the help of theoretical approaches, there are still some studies involving experimental evaluation of the elastic properties of N-CNTs. Arenal et al. [74] calculated the Young’s modulus of single-walled boron nitride nanotubes (SWBNNTs) from results of in situ uniaxial compression tests carried out by high-resolution transmission-electron microscopy (HRTEM) and atomic force microscopy (AFM). Chopra and Zettl [75] and Suryavanshi et al. [76] used a transmission electron microscope (TEM) to measure the Young’s modulus of multi-walled boron nitride nanotubes (MWBNNTs) from the thermal vibrational amplitude of a cantilevered nanotube and by the electric-field-induced resonance method, respectively. Golberg et al. [77] and Ghassemi et al. [78], with the help of AFM set up within TEM, performed in situ bending and cycling bending experiments, respectively, to evaluate the Young’s modulus of the MWBNNTs. Tanur et al. [79] studied the mechanical properties of MWBNNTs using a three-point bending technique in AFM and calculated their Young’s and shear moduli. Zhou et al. [80] measured the Young’s modulus of MWBNNTs using a high-order resonance technique within HRTEM. Chen et al. [81] studied the in situ mechanical behaviour of MWBNNTs under axial compression, using a TEM set up with a force transducer holder, and calculated the Young’s modulus of the MWBNNT, based on the directly measured critical compressive force. Hung et al. [16,82] studied the mechanical behaviour of single-walled gallium nitride nanotubes (SWGaNNTs) in compression using the nano-indentation technique and assessed their Young’s modulus and Poisson’s ratio. Stan et al. [15] performed experimental measurements of the Young’s modulus of faceted AlNNTs with triangular cross-section by contact resonance atomic force microscopy (CR-AFM).

The present review is focused on collecting and systematizing recent accomplishments in the mechanical characterization of inorganic non-carbon NTs, by numerical and analytical approaches. The outcomes achieved in the evaluation of the elastic constants (Young’s and shear moduli and Poisson’s ratio) and vibrational properties of the N-CNTs are examined.

## 2. Atomic Structure of N-CNTs

**Figure 1:**
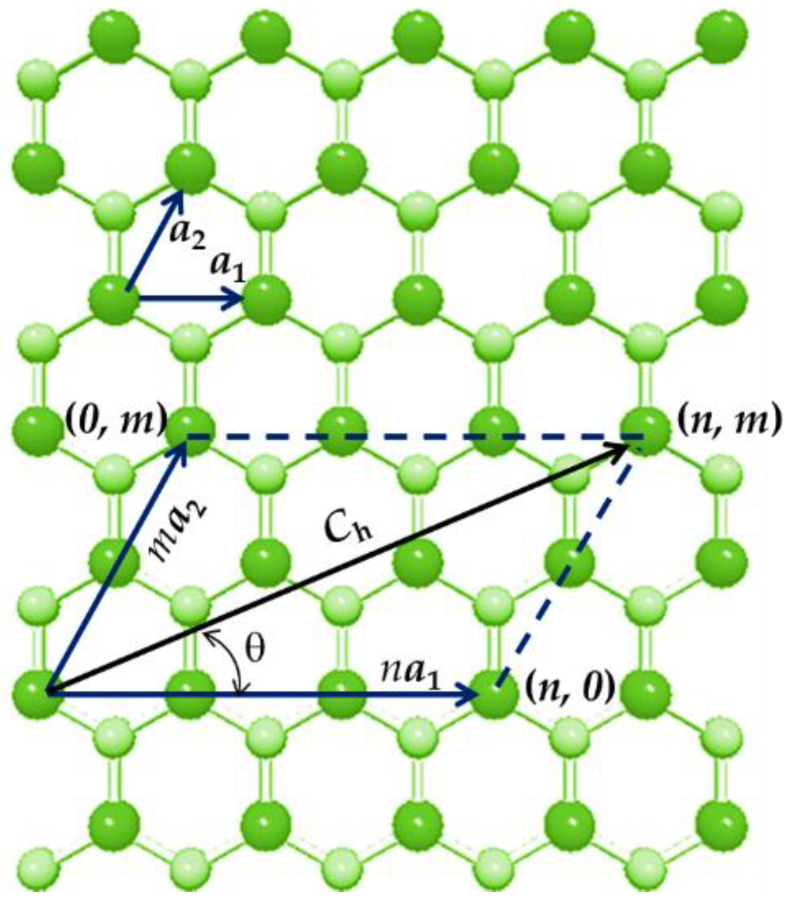
Representation of the hexagonal GaP lattice with designation of n and m, **C_h_** and θ. P atoms have a smaller radius and are represented in pale green and Ga atoms are in bright green.

Figure 1 shows how the parameters characterizing the atomic structure of non-carbon nanotubes are defined, namely the chiral indices, (n, m), the chiral vector, **C_h_**, and the chiral angle, θ, taking as an example a gallium phosphide (GaP) honeycomb lattice. Rolling up a hexagonal diatomic sheet into a cylinder results in the formation of non-carbon NT.

The chiral vector, **C_h_**, and the chiral angle, θ, are expressed in terms of the chiral indices, n and m as follows:

(1) $$C_{h}={na}_{1}+{ma}_{2},$$

(2) $${\theta =\sin}^{-1}\frac{\sqrt[{}]{3}}{2}\frac{m}{\sqrt[{}]{n^{2}{+\mathrm{nm}+m}^{2}}},$$

where $a_{1}$ and $a_{2}$ are the unit vectors of the hexagonal diatomic lattice, constituting atoms A1. and A2 and the chiral indices n and m are integers. The length of the unit vector a is defined as $a=\sqrt{3}a_{A1–A2}$, where $a_{A1–A2}$ is the equilibrium bond length. The bond length values for several diatomic nanostructures, which have been reported in the literature, are shown in Table 1. As can be seen, there is no agreement among the research community with regard to the bond length values for N-CNTs.

Considering the bond length, $a_{A1–A2}$, the N-CNT diameter, $D_{n}$ is defined as follows:

(3) $$D_{n}=\frac{a_{A1–A2}\sqrt{3\left(n^{2}{+\mathrm{nm}+m}^{2}\right)}}{\pi }.$$

Three main symmetry groups of N-CNTs are defined through the chiral angles, whose magnitudes are within the range of 0° to 30°, as follows:

- zigzag NTs (n, 0) when θ = 0° and m = 0;
- armchair NTs (n, n) when θ = 30° and n = m;
- chiral NTs (n, m) when 0° < θ < 30° and n ≠ m.

**Figure 2:**
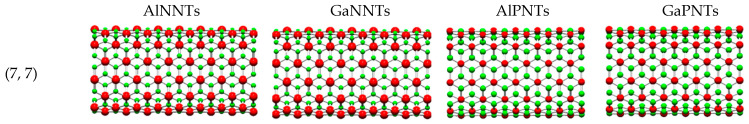

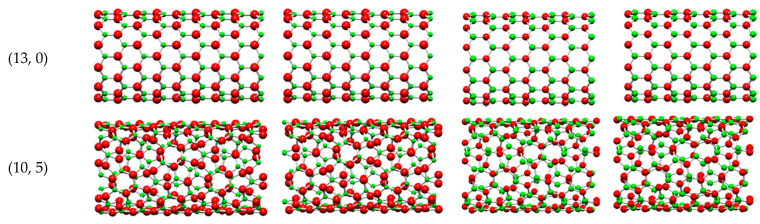
Illustration of the three main symmetry groups for AlN, GaN, AlP and GaP nanotubes with comparable diameters, constructed using the Nanotube Modeler© software. Al and Ga atoms are represented in red and N and P atoms are in green.

The two limiting configurations in terms of the chiral angle, zigzag (θ = 0°) and armchair (θ = 30°), are designated as non-chiral nanotubes. Segments of selected N-CNTs, representing three fundamental groups of symmetry, armchair, zigzag and chiral, are shown in Figure 2.

## 3. Analysis of the Literature Results

### 3.1. Elastic Constants of N-CNTs

#### 3.1.1. Young’s and Shear Moduli

Table 2 summarises theoretical results from the literature on the Young’s, E, and shear, G, moduli of different non-carbon NTs. Experimental results on the Young’s modulus evaluation of BNNTs and GaNNTs are also shown. Arenal et al. [74] calculated the Young’s modulus of SWBNNTs from the force—displacement curve obtained in the in situ uniaxial compression test of an individual SWBNNT, carried out in HRTEM and AFM set-ups. Hung et al. [16,82] also computed the Young’s modulus of SWGaNNTs with two different nanotube lengths, L_n_, from the force—displacement curve obtained in the uniaxial compression test performed using the Nano-indentation System (NS) with Berkovich indenter. Chopra and Zettl [75] examined a cantilevered MWBNNT by TEM and measured its Young’s modulus from the thermal vibrational amplitude, while Suryavanshi et al. [76] used, for the same propose, the electric-field-induced resonance method inside TEM. Zhou at al. [80] measured the Young’s modulus of the MWBNNTs using high-order resonance modes induced by electric fields, within HRTEM. Chen et al. [81], Golberg et al. [77] and Ghassemi et al. [78] calculated the MWBNNTs Young’s modulus in bending from experimentally obtained critical buckling force [81] and the force—displacement curves [77,78]. Tanur et al. [79] calculated the MWBNNTs Young’s and shear moduli from the results of the in situ three-point bending test, by AFM. The methodology of Stan et al. [15] to evaluate the Young’s modulus of the AlNNTs consisted of determining the contact stiffness between the AFM tip and the nanotube, combined with finite element analysis (FEA) calculations to take into account realistic contact area and, finally, to assess the modulus value from contact stiffness.

The elastic properties of the multi-walled (MW) nanotubes, i.e., the structures constituted by several single-walled NTs (layers) with the diameter of the outer layer, $D_{\mathrm{out}}$, were investigated in a few experimental works [75,76,77,78,79,80,81] in the case of MWBNNTs. It can be seen from this table that studies dealing with the Young’s modulus of N-CNTs are considerably more frequent than those on the evaluation of their shear modulus. The elastic moduli reported by analytical and numerical studies from the literature are obtained for the case of non-chiral (zigzag and armchair) N-CNTs. In the case of SWBNNTs, only Yan et al. [71] reported shear modulus values and Sakharova et al. [36] reported Young’s and shear moduli values. Pinhal et al. [29] calculated Young’s modulus for chiral SWAlNNTs and SWGaNNTs (see Table 2).

Since the calculation of the N-CNTs’ elastic moduli, E and G, almost always requires reliable knowledge of the value of the nanotube wall thickness, t_n_, several authors have provided the results concerning the surface Young’s ($E_{s}{=\mathrm{Et}}_{n}$) and shear ($G_{s}{=\mathrm{Gt}}_{n}$) moduli [33], this is taken into account in Table 2.

Although the InNNTs have been predicted theoretically [1,21], to our knowledge results regarding their mechanical properties are not available in the literature.

Table 2 shows that the Young’s modulus values obtained by theoretical methods for the BNNTs are in the range E ≈ 0.72–1.1 TPa, which is in reasonable agreement with the experimental results. Such resemblance of the elastic properties of the BNNTs to those of the CNTs (E ≈ 1.0 TPa) makes BNNTs suitable candidates either to replace CNTs in different technological applications [91,92], or to generate novel hybrid structures, based on carbon and boron-nitride NTs.

Moreover, it is evident from Table 2 that the studies to determine the shear modulus, G, of BNNTs are less common than those dealing with the evaluation of their Young’s modulus. The value of G is reported as half of that calculated for E in the respective studies [34,36,61,70,71]. Tanur et al. [79] in the only experimental study on this topic, as far we know, evaluated the shear modulus of MWBNNTs to be about 250 times smaller than their Young’s modulus. This low value was explained by the occurrence of shear between adjacent layers in the structure of the nanotube, due to the geometry and dimensions of the MWBNNTs under study.

With respect to the Young’s modulus, E, of other non-carbon NTs, only a few results were reported for AlNNTs and GaNNTs. The Young’s modulus of the AlNNTs, whose value is about half of that calculated for the BNNTs, was evaluated in the works of Hao et al. [47] (E ≈ 0.350 TPa), Pinhal et al. [29] (E ≈ 0.390 TPa), and Kang and Hwang [40] (E = 0.453 TPa). The results of Hao et al. [47] and Pinhal et al. [29] are in satisfactory agreement with those experimentally obtained for AlNNTs in the work of Stan et al. [15] (E = 0.325 TPa). A more considerable scatter of the Young’s modulus results can be observed for the GaNNTs. While Kang and Hwang [40] and Jeng et al. [38] obtained identical E values in their MD studies, equal to 0.796 TPa and 0.793 TPa, respectively, for (5, 5) GaNNT, Pinhal et al. [29] estimated by DFT calculations the Young’s modulus of (20, 20) GaNNT, equal to 0.383 GPa, and Hung et al. [16,82] in their experimental study using a nano-indentation test obtained the E values of 0.484 TPa and 0.223 TPa for the Young’s modulus of the SWGaNNTs with two different lengths.

In several works, only the surface Young’s modulus, E_s_, of the non-carbon nanotubes was evaluated. Based on the results of Baumeier et al. [46], Le [43], Genoese et al. [41] and Jiang and Guo [39], it can be concluded that the E_s_ value for the SiCNTs is about 40% lower than that calculated in the respective works for BNNTs. Kochaev [37] found that the surface Young’s moduli of AlNNTs, GaNNTs, AlPNTs and GaPNTs are approximately 30%, 40%, 50% and 70%, respectively, lower than the E_s_ value of BNNTs. Jiang and Guo [39] evaluated the surface Young’s moduli of AlNNTs, GaNNTs and BPNTs to be about 60% lower than that of BNNTs, and those of GaPNTs and InPNTs to be nearly 80% smaller than that of BNNTs. Although the studies concerning the evaluation of the mechanical properties of the N-CNTs other than BNNTs are rare, the preliminary results mentioned above point out that SiC, AlN, GaN, BP and, especially, AlP, GaP and InP nanotubes have low mechanical strength when compared with boron-nitride and carbon NTs. This should be taken into account when designing NT-based devices and hybrid nanostructures, where weaker N-CNTs are combined with other N-CNTs with high mechanical strength or CNTs.

**Figure 3:**
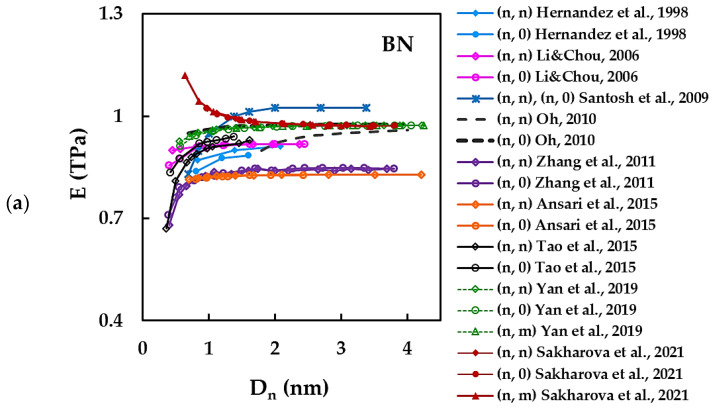

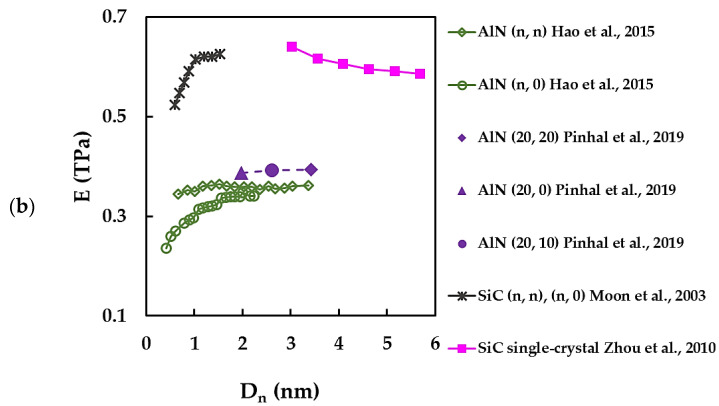
Young’s modulus, E, as a function of nanotube diameter, D_n_, for (**a**) BNNTs [34,36,56,59,60,61,62,66,71] and (**b**) AlNNTs [29,47] and SiCNTs [51,54].

In order to better analyse the evolutions of the Young’s modulus of the N-CNTs, the values of E in Table 2 were plotted, whenever it was possible, as a function of the nanotube diameter, $D_{n}$, in Figure 3a,b for the case of BNNTs (Figure 3a) and for the case of AlNNTs and SiCNTs (Figure 3b).

Discrepancies in the Young’s modulus values of BNNTs and in their evolutions can be observed in Figure 3. The Young’s modulus results for the BNNTs from Figure 3a, show three trends in the evolution of the value of E with the nanotube diameter, $D_{n}$:

- at first, the Young’s modulus decreases and then becomes almost stable for $D_{n}$ > 1.5 nm [36];
- the E value is nearly constant over the range of nanotube diameters [34,60,62,66,71];
- at first, the Young’s modulus increases and then becomes almost stable for 0.8 < $D_{n}$ < 1.5 nm [56,59,61].

It is worth noting that the Young’s modulus results obtained by Oh [62] for zigzag (n, 0) BNNTs, are in remarkable consonance with those evaluated by Yan et al. [71] for armchair (n, n), zigzag (n, 0) and chiral (n, m) BNNTs, over a wide range of $D_{n}$, despite different modelling approaches: CM by Oh [62] and longitudinal vibrations within NCM/MSM by Yan et al. [71]. Both Young’s modulus evolutions reported in the works of Oh and Yan et al. reasonably coincide with the E evolution obtained by Sakharova et al. [36] for armchair (n, n), zigzag (n, 0) and chiral (n, m) BNNTs with $D_{n}$ ≥ 1.715 nm. Satisfactory agreement is observed when comparing the Young’s modulus evolutions assessed by Zhang et al. [61] in their MD study and Ansari et al. [66], who used NCM/MSM approach, for (n, n) and (n, 0) BNNTs with $D_{n}$ ≥ 0.702 nm. A reasonable correspondence is observed between the Young’s modulus values evaluated by Tao et al. [56], who used the MD simulation, and Li and Chou [34], who employed the FE beam model under the NCM/MSM approach, for zigzag (n, 0) BNNTs. In most studies, the Young’s modulus evolution with nanotube diameter for (n, n) armchair BNNTs is separated from that for (n, 0) zigzag BNNTs [34,54,56,59,62,66], with the E values for armchair NTs being higher than those for zigzag NTs. Santos et al. [59] reported the same Young’s modulus evolution for both (n, n) and (n, 0) BNNTs. Yan et al. [71] and Sakharova et al. [36] found that the evolutions of E with $D_{n}$ are described by a unique trend for armchair, zigzag and chiral nanotubes.

As can be seen from Figure 3b, the evolutions of the Young’s modulus as a function of the nanotube diameter, $D_{n}$, reported by Moon et al. [51] for the SiCNTs, shows a unique trend for (n, n) and (n, 0) zigzag nanotubes, where E increases for small $D_{n}$ and then tends to a nearly constant value. On the contrary, the E value of the monocrystalline SiCNTs slightly decreases at the beginning and then becomes stable with increasing $D_{n}$ [54]. In the case of AlNNTs, the Young’s modulus evolutions in nanotube diameter can be separated for (n, n) armchair and (n, 0) zigzag NTs, as reported by Hao at al. [47] (see Figure 3b). The E value of (n, n) AlNNTs is almost constant for all range of $D_{n}$, while E of (n, 0) AlNNTs substantially increases for $D_{n}$ ≲ 1.554 nm and after that it stabilizes. The E values obtained by Hao at al. [47] for (n, n) AlNNTs are about 9% lower than those calculated by Pinhal et al. [29] for individual (20, 20), (20, 0) and (20, 10) AlNNTs.

**Figure 4:**
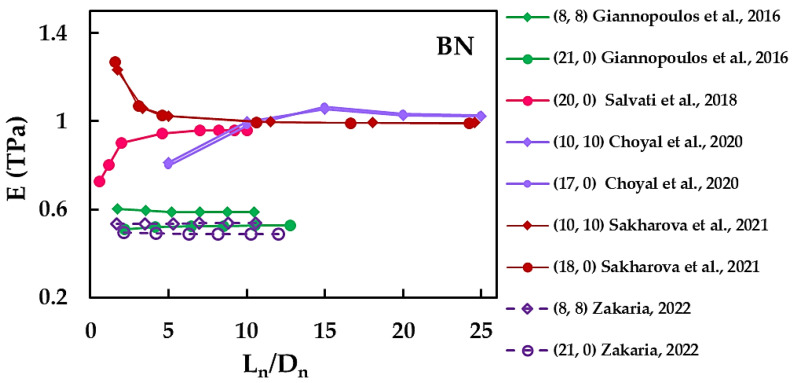
Young’s modulus, E, of BNNTs as a function of the nanotube aspect ratio, $L_{n}/D_{n}$ [31,36,65,68,69].

To further analyse, the Young’s modulus results of the BNNTs in Table 2, were plotted as a function of the nanotube aspect ratio, $L_{n}$/$D_{n}$, and the nanotube diameter, $D_{n}$ (see Figure 4).

/$D_{n}$ [31,36,65,68,69].

The E evolutions as a function of $L_{n}$/$D_{n}$ reported by Sakharova et al. [36] for (10, 10) and (18, 0) BNNTs are in satisfactory agreement with those obtained by Choyal et al. [31] for (10, 10) and (17, 0) BNNTs and by Salvati et al. [65] for (20, 0) BNNT, in case of nanotube aspect ratios $L_{n}$/$D_{n}$ > 8. Zakaria [68] and Giannopoulos et al. [69], in their numerical simulation studies within NCM/MSM approach, obtained for (8, 8) and (21, 0) BNNTs Young’s modulus values similar in all range of the nanotube aspect ratio (see, Figure 4), with differences of less than about 8% in the E values. Note that, despite using dissimilar methods to model the B–N covalent bond, Giannopoulos et al. [69] used spring elements and Zakaria [68] represented the interatomic bond as two-section beam, in both works the elastic properties of the elements to be used as input for the numerical simulation were computed based on the same values of the constants k_r_, k_θ_ and k_τ_.

**Figure 5:**
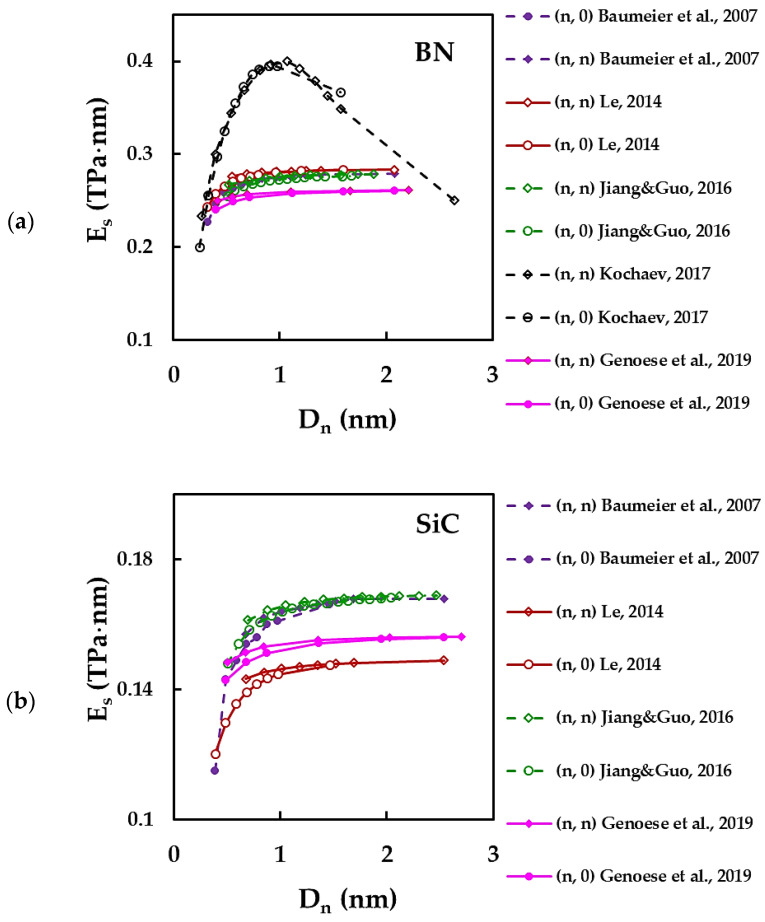

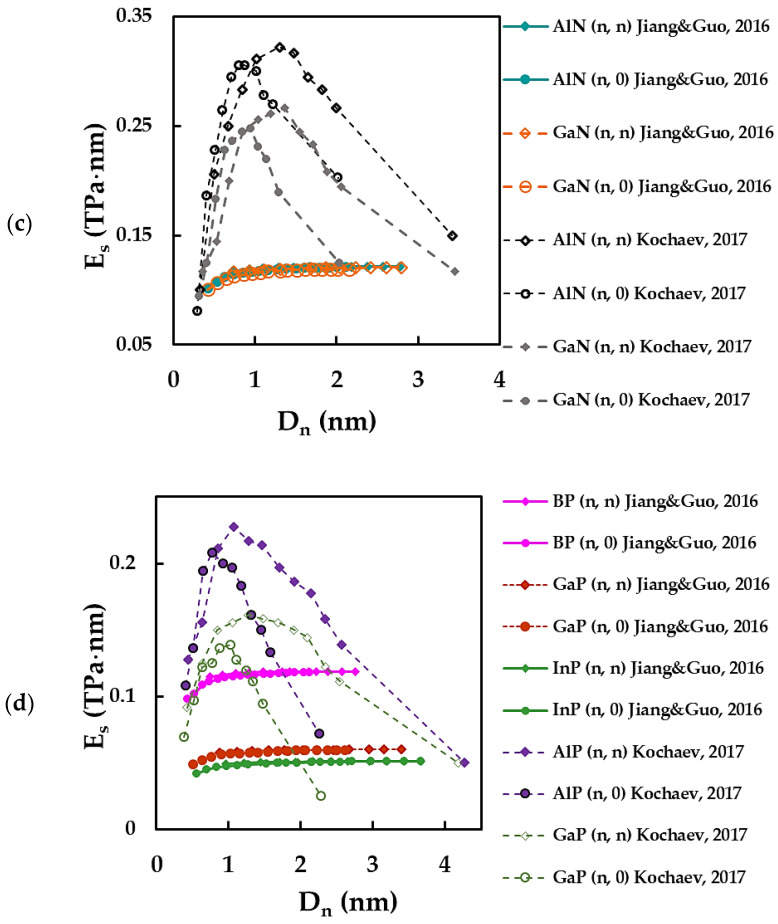
Surface Young’s modulus, $E_{s}$, as a function of the nanotube diameter, $D_{n}$, for (**a**) BNNTs [37,39,41,43,46], (**b**) SiCNTs [39,41,43,46], (**c**) AlN [37,39], GaNNTs [37,39], (**d**) AlP [37], GaP [37,39], BP [39], InPNTs [39].

Figure 5 shows the evolution of the surface Young’s modulus, $E_{s}$, with the nanotube diameter, $D_{n}$, for BNNTs (Figure 5a), SiCNTs (Figure 5b), AlN, GaNNTs (Figure 5c), and BP, AlP, GaP, InPNTs (Figure 5d). Regarding Figure 5a,c,d, it can be noted that there are two characteristic trends of the evolution of the surface Young’s modulus, $E_{s}$, with the nanotube diameter, $D_{n}$, one for the nitrides (BN, AlN, GaN) and another for phosphides (BP, AlP, GaP, InP). In the first case, the $E_{s}$ value slightly increases with increasing of $D_{n}$, and then becomes nearly constant for nanotube diameters $D_{n}$ ≳ 0.7 nm [39,41,43,46]. The second trend is reported by Kochaev [37] for BN, AlN, GaN, AlP, GaPNTs, consists of a significant increase of the surface Young’s modulus for $D_{n}$ ≲ 1.2 nm (armchair NTs) and $D_{n}$ ≲ 0.9 nm (zigzag NTs), and then a strong decrease of $E_{s}$ after reaching the maximum.

In the case of SiC, the only representative of carbide NTs in the present analysis, the $E_{s}$ evolution follows a trend in which its value increases for small nanotube diameters, $D_{n}$, of less than 0.7 nm, and remains approximately constant for large diameters (Figure 5b).

Concerning the BNNTs, Le [43], Baumeier et al. [46] and Jiang and Guo [39] provided obtained comparable $E_{s}$ results (see, Figure 5a), Genoese et al. [41], employing “stick-and-spring” model similar to that used by Jiang and Guo [39], evaluated the values of $E_{s}$ at about 6% lower than those reported by the latter. Such difference is probably related to the approaches used for computation of the force-field constants. The maximum value of the surface Young’s modulus obtained by Kochaev [37] for BNNTs is notably higher than the values calculated by other authors [39,41,43,46]. The same is true for the surface Young’s modulus results of AlN, GaN and GaP nanotubes, when compared with the values reported by Kochaev [37] and Jiang and Guo [39] (see, Figure 5c,d). Jiang and Guo [39] found that the $E_{s}$ values of armchair and zigzag structures are approximately identical for AlN, GaN, BP, GaP, and InPNTs. Regarding the surface Young’s modulus of SiCNTs, there is a good agreement between the results reported by Baumeier et al. [46] and Jiang and Guo [39]. The values of $E_{s}$ calculated by Genoese et al. [41] and Le [43] are, respectively, about 7% and 11% lower than those of Baumeier et al. [46] and Jiang and Guo [39], while the $E_{s}$ value assessed by Setoodeh et al. [52] is about 8% higher (see, Figure 5b).

**Figure 6:**
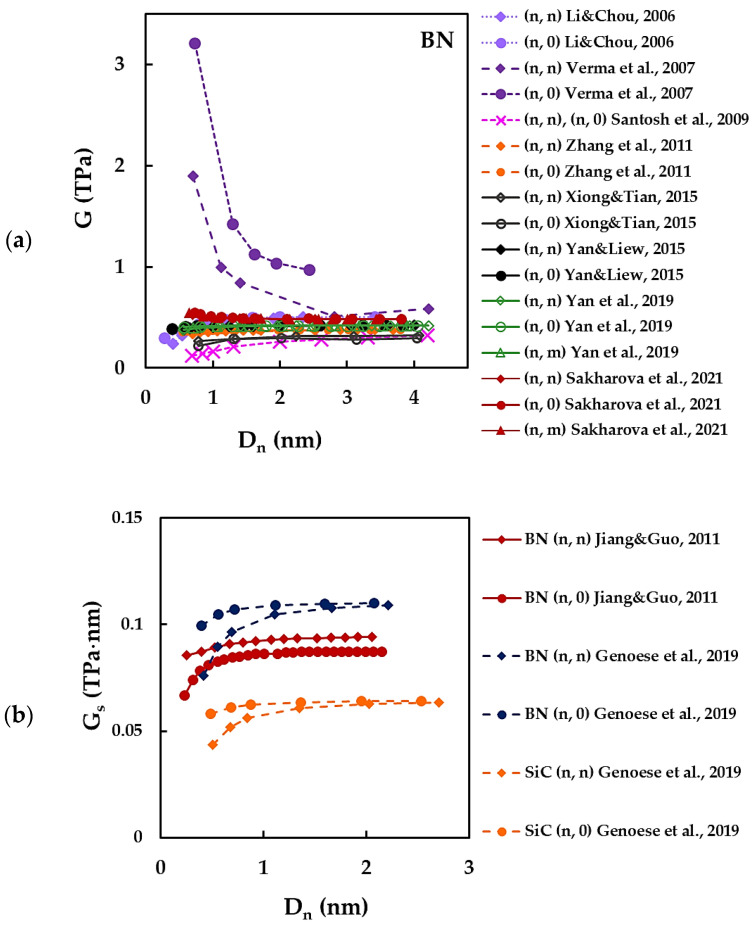
(**a**) Shear modulus, G, for BNNTs [34,36,50,55,59,61,70,71] and (**b**) surface shear modulus, $G_{s}$, for BNNTs [33,41] and SiCNTs [41], as a function of the nanotube diameter, $D_{n}$.

The results of Table 2 concerning the evolutions of the shear, G, and surface shear, $G_{s}$, moduli with the nanotube diameter, $D_{n}$, are shown in Figure 6a,b, respectively. With an exception of the $G_{s}$ values for SiCNTs reported by Genoese et al. [41], all other shear and surface shear moduli results plotted in Figure 6 are related to BNNTs. As in the case of the Young’s modulus, three trends of the evolution of G and $G_{s}$ with $D_{n}$ can be found in the literature:

- at the beginning, the shear modulus decreases along with increase in the diameter of the BNNTs and then tends to stabilize at high values of $D_{n}$ [36,55];
- the shear modulus almost does not vary within the entire range of BNNT diameters [61,70,71];
- at the beginning, the shear modulus slightly increases and then becomes nearly constant for high values of $D_{n}$ [33,34,41,50,59].

Most authors, such as Li and Chou [34], Sakharova et al. [36], Zhang et al. [61], Yan and Liew [70] and Yan et al. [71] pointed to the value of 0.426 ± 0.06 TPa as the shear modulus of BNNTs (see, Figure 6a). In these works [34,36,61,70,71], the G values for (n, n) armchair, (n, 0) zigzag and (n, m) chiral structures (when those are reported, as for example [36,71]) are also practically identical for nanotube diameters $D_{n}$ > 0.8 nm.

Santos et al. [59] and Xiong and Tian [50] reported similar G values for BNNTs, especially for nanotube diameters $D_{n}$ > 2 nm (see, Figure 6a). The shear modulus values obtained by Santos et al. [59] and Xiong and Tian [50] are about 25% lower than those presented [34,36,61,70,71] (see, Figure 6b).

With regard to surface shear modulus, Genoese et al. [41] reported the value of $G_{s}$ of SiCNTs at about 40% lower than that of BNNTs. In the work of Jiang and Guo [39], the surface shear modulus of the zigzag structure of SiCNTs and BNNTs are higher when compared to those of the armchair structure for $D_{n}$ ≲ 1.95 nm. The $G_{s}$ values evaluated by Jiang and Guo [39] are lower than those obtained by Genoese et al. [41]. These studies share the modelling approach, but the methods for calculating the force-field constants and the surface shear modulus are different, which could explain the discrepancy of the results. Jiang and Guo [39] introduced the out-of-plane inversion force constant to describe the bond torsion, which is essential to calculate the shear modulus, while Genoese et al. [41] assumed a continuum thin shell model to assess $G_{s}$.

It is worth noting that the scattering observed in the values of the elastic modulus of the N-CNTs can be attributed to the different modelling and calculation methods used. The main limitation of the NCM/MSM approach is related to accurate determination of the force field constants. Although knowledge of these constants is not necessary to employ the atomistic approach, the latter is generally time consuming and computationally expensive. Another restriction to the calculation of the elastic moduli is associated with the value of the nanotube wall thickness, $t_{n}$. Except for the case of BNNTs, there is an apparent lack of information in the literature about the value of $t_{n}$ for other N-CNTs. For this reason, the surface elastic moduli of N-CNTs, different from BNNTs, were mainly assessed, which makes it difficult to compare the available results.

#### 3.1.2. Poisson’s Ratio

The results of the Poisson’s ratio of N-CNTs available in the literature are summarized in Table 3. The only experimental value in the table was obtained by Hung et al. [16,82] in the uniaxial compression test performed with the help of the nano-indentation system (NS) for SWGaNNTs.

As can be seen in Table 3, the Poisson’s ratio values for GaNNTs evaluated by Jeng et al. [38], in their MD study, and by Jiang and Guo [39], using the analytical model under NCM/MSM approach, are in acceptable agreement with the ν value calculated by Hung et al. [16] from the experimental results of the nano-indentation test. The difference between the values of ν reported by Jeng et al. [39] and Jiang and Guo [39] and those experimentally obtained [16] is around 8% and 15%, respectively.

**Figure 7:**
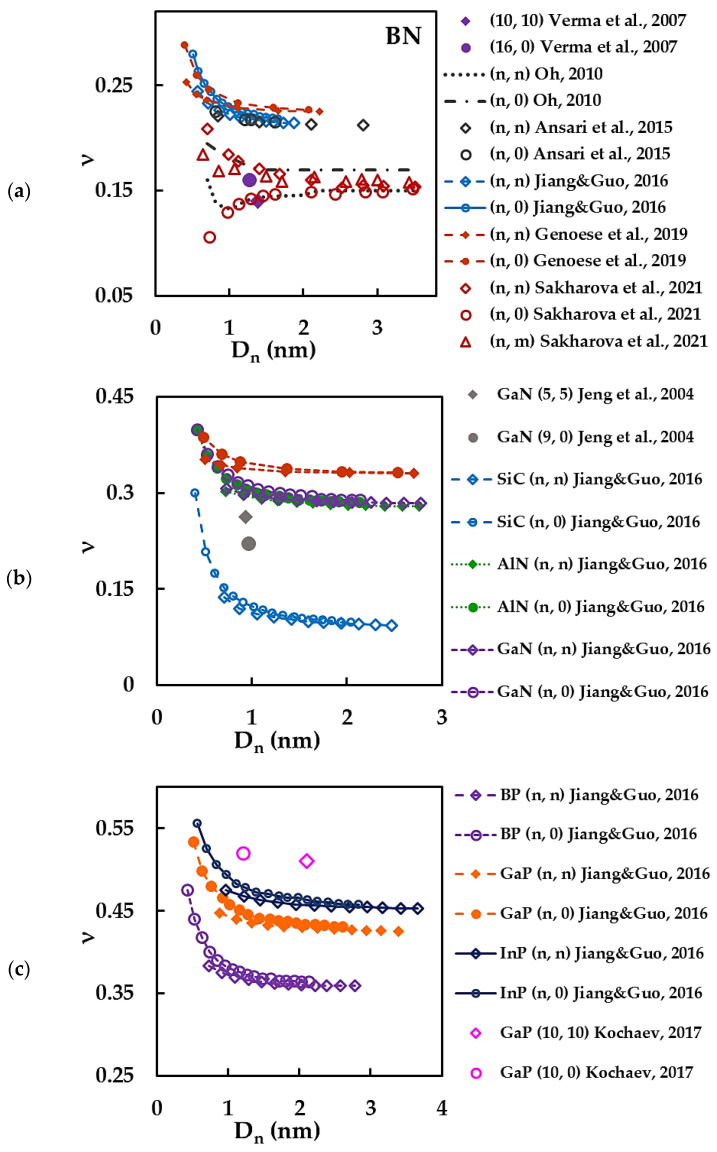
Poisson’s ratio, ν, as a function of the nanotube diameter, $D_{n}$, for (**a**) BNNTs [36,39,41,55,62,66], (**b**) SiC [39,41], AlN [39], GaNNTs [38,39] and (**c**) BP [39], GaP [37,39], InPNTs [39].

To facilitate the analysis of the literature results from Table 3, the Poisson’s ratio of N-CNTs was plotted as a function on the nanotube diameter, $D_{n}$, as shown in Figure 7. The stabilized values of Poisson’s ratio found in the literature for BNNTs can be separated into two groups (Figure 7a). Jiang and Guo [39], Genoese et al. [41] and Ansari et al. [66] obtained the values of ν in the range of 0.217 to 0.239, for the nanotube diameter $D_{n}$ ≳ 1.2 nm. The correspondent evolutions of the ν value with $D_{n}$ show that the Poisson’s ratio decreases for small BNNT diameters and tends to stabilize with the increase of $D_{n}$, the evolutions of ν reported by Jiang and Guo [39] for (n, n) armchair, and Ansari et al. [66] for (n, n) armchair and (n, 0) zigzag BNNTs, being in especially good agreement (see, Figure 7a). For all the above mentioned results, the ν value for (n, 0) nanotubes is higher than that obtained for (n, n) nanotubes.

Sakharova et al. [36], Oh [62] and Verma et al. [55] evaluated the BNNT Poisson’s ratio in the range of 0.14–0.17 for nanotubes with diameter $D_{n}$ ≳ 1.2 nm (see, Figure 7a). Regarding trends in the evolution of ν with $D_{n}$, Sakharova et al. [36] reported that for $D_{n}$ < 1.5 nm the Poisson’s ratio value decreases for the cases of (n, n) armchair and (n, m) chiral BNNTs, but increases in the case of (n, 0) zigzag BNNTs. When the nanotube diameter becomes higher than 1.5 nm, ν tends to a nearly constant value for (n, n), (n, 0) and (n, m) BNNTs.

Thus, according to Sakharova et al. [36], for BNNT diameters $D_{n}$ < 1.5 nm, the value of ν is clearly influenced by the chiral angle of the nanotube and increases from zigzag structure (θ = 0°) to armchair (θ = 30°). Nevertheless, Oh [62] found that the Poisson’s ratio of both BNNTs, (n, n) armchair and (n, 0) zigzag, decreases for $D_{n}$ < 1.5 nm and afterwards becomes constant, the value of ν being greater for the (n, 0) structures.

Concerning N-CNTs different from BNNTs, it can be concluded from Figure 7b,c that the evolutions of the Poisson’s ratio, ν, with nanotube diameter, $D_{n}$, follow a similar trend, for which the ν value decreases for small nanotube diameters and then tends to stabilize with increasing $D_{n}$ (cases of SiC, AlN, GaN, BP, GaP and InPNTs). For all the above mentioned evolutions of the ν value with $D_{n}$, ν is higher for (n, 0) zigzag NTs. Jiang and Guo [39] established, in their study for AlN, GaN, BP, GaP and InPNTs, that the greater the value of the bond length, $a_{A1–A2}$, of the diatomic hexagonal nanostructure (see, Table 1), the greater the value of the nanotube diameter, $D_{n}$, for which the Poisson’s ratio becomes stable (see, Figure 7b,c).

Genoese et al. [41] reported the value of ν for SiCNTs to be about 3.5 times higher than that calculated by Jiang and Guo [39] (see, Figure 7b). Such a large scatter in the ν values cannot be attributed solely to the different methods for computation of the force field constants, under a similar modelling approach in both studies, because the results of the Poisson’s ratio for BNNTs, which were also obtained in these studies, do not show such considerable dissimilarity (see, Figure 7a). In fact, Jiang and Guo [39] calculated the force field constants for BN and SiC nanotubes with the same calculation method. Genoese et al. [41] took into account the assumption of the so-called buckled surface of SiC nanotube, which occurs due to the fact that Si and C atoms form two coaxial cylinders [46,93,94]. This hypothesis modified the way of calculating the force field constants for SiCNTs compared to BNNTs.

The results of Jiang and Guo [39] obtained for other N-CNTs can be compared only for the cases of GaNNTs and GaPNTs, reported by Jeng et al. [38] and Kochaev [37], respectively. Jeng et al. [38] obtained the Poisson’s ratio values for (5, 5) and (9, 0) GaNNTs 12% and 29% lower, respectively, than those evaluated by Jiang and Guo [39] for GaNNTs with comparable diameters. In the work by Kochaev [37], the Poisson’s ratio of (10, 10) and (10, 0) GaPNTs is 19% and 17% higher, respectively, than the value of ν estimated by Jiang and Guo [39], for GaP nanotubes with comparable diameters.

It can be noted that there is large scattering of the ν values reported in the literature for N-CNTs, regardless of the modelling and calculation approaches used for this purpose.

### 3.2. Vibtational Properties of N-CNTs

The developing of innovative nanoelectromechanical devices, such as oscillators, amplifiers, mass/charge detectors ultra-high frequency resonators and resonant-based nano-mechanical sensors [95,96,97], as well their top-notch performance, requires constituents with appropriate vibrational characteristics. The N-CNTs, in particular the BNNTs, offer the necessary properties for nanoscale electromechanical applications, specifically sensing ones. In this context, understanding the dynamical properties of the N-CNTs is crucial for creating novel nano-devices with desirable functioning.

The studies reported so far on the dynamic properties of N-CNTs mainly addressed the theoretical analysis of the vibrational behaviour of BNNTs [34,48,57,63,67,68,69,70,71,98,99] and SiCNTs [100,101].

Table 4 summarizes the available theoretical results on the vibrational properties of N-CNTs. Since in general the works deal with the calculation of the fundamental frequencies of nanotubes, the first fundamental frequency, *f*_n1_, is herein chosen for the comparison purpose. The vibration analysis of NTs is typically performed assuming different support constraints, depending on their potential applications, i.e., simply supported (SS), clamped-free (CF) and clamped-clamped (CC) boundary conditions, which are indicated in Table 4.

**Figure 8:**
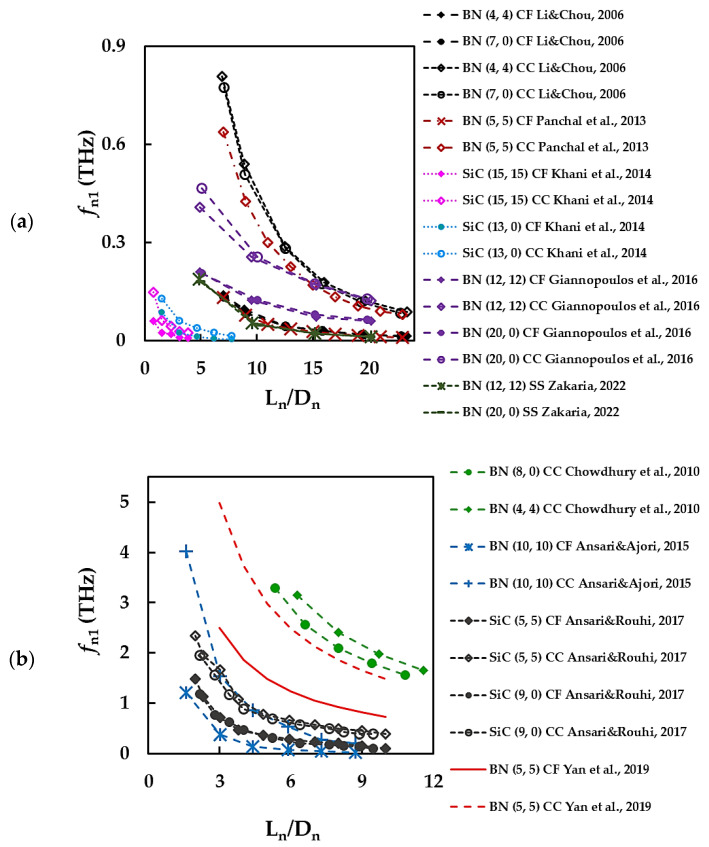
The first fundamental frequency, *f*_n1_, as a function of the NT aspect ratio, $L_{n}$/$D_{n}$ [34,57,67,68,69,71,98,100,101]; the *f*_n1_ values of comparable magnitude are plotted on the different graphs (**a**,**b**).

**Figure 9:**
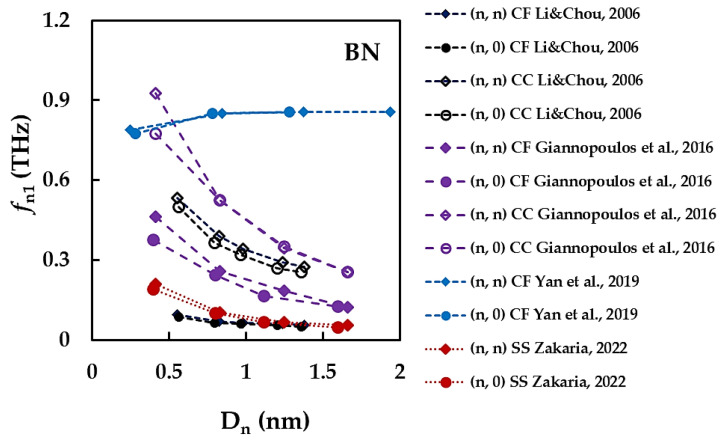
The first fundamental frequency, *f*_n1_, as a function of the nanotube diameter, $D_{n}$, for BNNTs [34,68,69,71].

In order to simplify the understanding of the results from Table 4, the first fundamental frequency, *f*_n1_, was plotted as a function of the NT aspect ratio, $L_{n}$/$D_{n}$, and NT diameter, $D_{n}$, in Figure 8 and Figure 9, respectively. It can be concluded from Figure 8a,b and Table 4 that the values of *f*_n1_ are higher for the case of clamped-clamped (CC) boundary conditions than for the case of clamped-free (CF) boundary conditions [34,57,67,69,71,100]. The first fundamental frequency decreases with the nanotube aspect ratio and the decreasing rate is higher when the clamped-clamped boundary conditions is applied. Regarding the *f*_n1_ value of BNNTs, the results reported by Li and Chou [34] and Panchal et al. [67] for clamped-free support, and by Zakaria [68] for simply supported NTs are in a particularly good agreement for all range of the aspect ratio, $L_{n}$/$D_{n}$, considered (see Figure 8a).

The values of *f*_n1_ obtained by Giannopoulos et al. [69] under clamped-free boundary conditions are about 30% higher when compared with *f*_n1_ calculated in the abovementioned studies [34,67,68] for $L_{n}$/$D_{n}$ ≥ 10. The *f*_n1_ values reported by Li and Chou [34], Panchal et al. [67] and Giannopoulos et al. [69], considering clamped-clamped boundary conditions, are in satisfactory agreement with each other for $L_{n}$/$D_{n}$ ≥ 15. For the rest of the results from Table 4 and Figure 8, there is a considerable scattering of the *f*_n1_ values for both BNNTs and SiCNTs. Although the representation of *f*_n1_ as a function of the nanotube diameter, $D_{n}$, is less frequent, some results for the BNNTs can be found in the literature, which are shown in Figure 9.

The main trend of the evolution of the first fundamental frequency with the nanotube diameter is the decrease of *f*_n1_ when $D_{n}$ increases, whatever the boundary conditions [34,68,69]. According to another trend, reported by Yan et al. [71], the *f*_n1_ value slightly increases for nanotube diameters $D_{n}$ < 0.7 nm and then is almost constant with increasing D_n_.

It is worth mentioning that most of the reported results on dynamic properties of the N-CNTs are limited to the vibrational analysis of some BNNTs and the determination of their first fundamental frequency. That is, the vibrational properties of N-CNTs are less commonly investigated than their elastic moduli and Poisson ratio. In this context, considering forthcoming applications, systematic studies are needed on the vibrational behaviour of a wider set of N-CNTs with a wide range of chiral indices and diameters.

## 4. Conclusions

The present review collects accomplishments in the development of the modelling of elastic properties of N-CNTs. Works in the literature, where the static (Young’s and shear moduli, and Poisson’s ratio) and dynamic (first fundamental frequency) elastic properties were evaluated, are comprehensively analysed. Despite considerable achievements in predicting the elastic properties of BNNTs by numerical simulation and analytical approaches, the other N-CNTs have received much less research attention. Theoretical studies show scattering in the values of elastic constants and the first fundamental frequency due to different modelling and calculation approaches. Most of the results on the elastic properties of N-CNTs were obtained using the atomistic and NCM/MSM approaches. Although the NCM/MSM approach proves to be cost-effective for modelling the mechanical behaviour of N-CNTs, the main challenge in its application remains the suitable choice of the force field constants to simulate a bond between two atoms in the diatomic nanotube structure. If for the BNNTs this choice is ambiguous, the methods for computing the force field constants for other N-CNTs are scarce and not well explored.

In this context, future lines of research on the evaluation of the elastic properties of N-CNTs should include the development of the NCM/MSM approach, focusing on the correct computation of the force field constants. It is expected that the upcoming investigation will provide a benchmark regarding the determination of the mechanical properties of a wide group of N-CNTs by theoretical (numerical and analytical) methods.

## Figures and Tables

**Table 1:** Values of the bond length of hexagonal diatomic nanostructures available in the literature.

Compound	BN	AlN	GaN	InN	BP	AlP	GaP	InP	SiC
**a_A1–A2_, nm**	0.1447 [83] 0.145 [84] 0.147 [37] 0.151 [85] 0.153 [33]	0.177 [37] 0.179 [84] 0.185 [40] 0.193 [86] 0.195 [87]	0.175 [88] 0.184 [37] 0.185 [84] 0.186 [40] 0.194 [86]	0.203 [1] 0.206 [84]	0.183 [84] 0.193 [86]	0.234 [89] 0.240 [37]	0.220 [37] 0.225 [84] 0.229 [90] 0.236 [86]	0.246 [84] 0.256 [86]	0.177 [84] 0.179 [51] 0.185 [86]

**Table 2:** Young’s and shear moduli results for non-carbon nanotubes reported in the literature.

Approach	Year	Reference	Method	Type of NTs ^1^		E, TPa ^2^	E_s_, TPa⋅nm	G, TPa ^2^	G_s_, TPa⋅nm	Comment
Atomistic	1998	Hernandez et al. [60]	TBMD	BN	(n, n)	0.894	–	–	–	average value
					(n, 0)	0.896				
	2003	Moon et al. [51]	MD: Tersoff empirical potential	SiC	(n, n)	0.621	–	–	–	average value
					(n, 0)	0.558				
	2004	Jeng et al. [38]	MD: TB many body potential	GaN	(5, 5)	0.793	–	–	–	–
					(9, 0)	0.721				
	2004	Kang and Hwang [40]	MD: Tersoff-type potential	BN	(5, 5)	0.870	–	–	–	–
				AlN		0.453				
				GaN		0.796				
	2007	Baumeier et al. [46]	ab initio: DFT-SIC	BN	(n, n)	–	0.278	–	–	converged average value
					(n, 0)		0.272			
				SiC	(n, n)		0.167			
					(n, 0)		0.162			
	2007	Verma et al. [55]	MD: TB potential	BN	(n, n)	1.107	–	0.965	–	average value
					(n, 0)	1.044		1.555		
	2009	Santosh et al. [59]	MD: force—constant approach	BN	(n, n); (n, 0)	1.017	–	0.326	–	converged average value
	2009	Setoodeh et al. [52]	MD: Tersoff potential	SiC	(n, n)	–	0.182		–	average value
					(n, 0)		0.180			
	2009	Pan and Si [53]	MD: Tersoff bond order potential	SiC	single crystalline	0.465	–	–	–	t_n_ = 0.30 nm
						0.540				t_n_ = 0.90 nm
	2010	Zhou et al. [54]		SiC		0.641	–	–	–	t_n_ = 0.89 nm
						0.595				t_n_ = 1.69 nm
						0.582				t_n_ = 2.49 nm
	2011	Zhang et al. [61]	MD: DFTB	BN	(n, n)	0.840	–	0.366	–	converged average value
					(n, 0)	0.844		0.368		
	2014	Le [43]	MD: harmonic force fields	BN	(n, n)	–	0.282	–	–	converged average value
					(n, 0)		0.281			
				SiC	(n, n)		0.148			
					(n, 0)		0.145			
	2015	Hao et al. [47]	ab initio: LCAO	AlN	(n, n)	0.360	–	–	–	converged average value
					(n, 0)	0.340				
	2015	Xiong and Tian [50]	MD, Tersoff potential: force approach	BN	(n, n)	–	–	–	0.315	average value
					(n, 0)				0.329	
			energy approach		(n, n)				0.281	
					(n, 0)				0.292	
	2015	Tao et al. [56]	MD: TB potential + FE model	BN	(n, n)	0.911	–	–	–	converged average value
					(n, 0)	0.930				
	2017	Kochaev [37]	ab initio	BN	(n, n)	–	0.347	–	–	average value
					(n, 0)		0.340			
				AlN	(n, n)		0.253			
					(n, 0)		0.247			
				GaN	(n, n)		0.207			
					(n, 0)		0.193			
				AlP	(n, n)		0.172			
					(n, 0)		0.159			
				GaP	(n, n)		0.131			
					(n, 0)		0.106			
	2019	Pinhal et al. [29]	DFT + B3LYP	AlN	(20, 20)	0.393	–	–	–	–
					(20, 0)	0.387				
					(20, 10)	0.392				
				GaN	(20, 20)	0.383				
					(20, 0)	0.367				
					(20, 10)	0.370				
	2020	Choyal et al. [31]	MD: TB potential	BN	(10, 10)	1.053	–	–	–	L_n_ ≈ 21 nm
					(17, 0)	1.066				
	2020	Vijayaraghavan and Zhang [35]	MD: REBO	BN	(10, 10)	2.8	–	–	–	t_n_ = 0.105 nm
CM	2010	Oh [62]	CL thermodynamic approach + TB potential	BN	(n, n)	0.960	–	–	–	converged average value
					(n, 0)	0.975				
NCM/MSM	2006	Li and Chou [34]	beams + FE model	BN	(n, n)	0.916	–	0.465	–	converged average value
					(n, 0)	0.913		0.475		
	2011	Jiang and Guo [33]	“stick-and-spring” model + closed-form solution	BN	(n, n)		0.270		0.095	converged average value
					(n, 0)		0.262		0.088	
	2015	Ansari et al. [66]	analytical solution	BN	(n, n)	0.825	–	–	–	average value
					(n, 0)	0.823				
	2015	Yan and Liew [70]	representative cell	BN	(n, n)	0.970	–	0.416	–	converged average value
					(n, 0)	0.967		0.418		
	2016	Giannopoulos et al. [69]	springs + FE model: free vibrations	BN	(12, 12)	0.592	–	–	–	L_n_ ≈ 11 nm
					(21, 0)	0.523				
	2016	Jiang and Guo [39]	“stick-and-spring” model + analytical	BN	(n, n)	–	0.278	–	–	converged average value
					(n, 0)		0.276			
				AlN	(n, n)		0.121			
					(n, 0)		0.120			
				GaN	(n, n)		0.120			
					(n, 0)		0.119			
				BP	(n, n)		0.118			
					(n, 0)		0.117			
				GaP	(n, n)		0.060			
					(n, 0)		0.059			
				InP	(n, n)		0.051			
					(n, 0)		0.051			
				SiC	(n, n)		0.169			
					(n, 0)		0.168			
	2018	Salavati et al. [65]	beams + FE model	BN	(n, n); (n, 0)	0.928	–	–	–	converged average value
	2019	Yan et al. [71]	longitudinal and torsional vibrations	BN	(n, n); (n, 0); (n, m)	0.972		0.418	–	converged average value
	2019	Genoese et al. [41]	“stick-and-spring” model + Donnell thin shell model	BN	(n, n)	–	0.255	–	0.092	converged average value
					(n, 0)		0.250		0.104	
				SiC	(n, n)		0.152		0.053	
					(n, 0)		0.149		0.061	
	2021	Sakharova et al. [36]	beams + FE model	BN	(n, n); (n, 0); (n, m)	0.984	–	0.486	–	converged average value
	2022	Zakaria [68]	two-section beams + FE model	BN	(12, 12)	0.538		0.108		L_n_ ≈ 11 nm
					(21, 0)	0.489		0.117		
Experimental	1998	Chopra and Zettl [75]	TEM: thermal vibrational amplitude	MWBNNTs		1.220 ± 0.240	–	–	–	–
	2004	Suryavanshi et al. [76]	TEM: electric-field-induced resonance	MWBNNTs with $D_{\mathrm{out}}$ = 34–94 nm		0.722 ± 0.140	–	–	–	average value for 18 MWBNNTs
	2005	Hung et al. [16]	NS + analytical	SWGaNNTs		0.484	–	–	–	L_n_ = 500 nm
						0.223				L_n_ = 300 nm
	2007	Goldberg et al. [77]	AFM-TEM: bending + analytical	MWBNNTs with $D_{\mathrm{out}}$ = 40–100 nm		0.5–0.6	–	–	–	average value
	2009	Stan et al. [15]	CR-AFM + FEA	faceted AlNNTs with triangular cross-section		0.3252 ± 0.015	–	–	–	inner facet
						0.3050 ± 0.013				outer facet
	2010	Ghassemi et al. [78]	AFM-TEM: bending + analytical	MWBNNTs with $D_{\mathrm{out}}$ = 38–51 nm		0.5 ± 0.1	–	–	–	average value for 5 NTs
	2011	Arenal et al. [74]	HRTEM-AFM + analytical	SWBNNTs		1.11±0.17	–	–	–	t_n_ = 0.07 nm
						0.87±0.13				t_n_ = 0.09 nm
						0.25±0.04				t_n_ = 0.34 nm
	2013	Tanur et al. [79]	AFM: a three-point bending + analytical	MWBNNTs with $D_{\mathrm{out}}$ = 18–55 nm		0.760 ± 0.03	–	0.07 ± 0.01	–	E in bending, average value (0.1 ± 0.02 to 1.8 ± 0.3 TPa) for 20 NTs
	2019	Zhou et al. [80]	HRTEM: high-order resonance	MWBNNTs with $D_{\mathrm{out}}$ = 28–57 nm		0.906	–	–	–	average value
	2019	Chen et al. [81]	TEM: force transducer holder + analytical	MWBNNT with $D_{\mathrm{out}}$ = 37.34 nm and 40 layers		1.050–1.370	–	–	–	E calculated from tree compression cycles

^1^ All theoretical results presented were obtained for single-walled NTs. ^2^ The Young’s, E, and shear, G, moduli values for the BNNTs were evaluated, considering the nanotube wall thickness, t_n_ ≈ 0.34 nm; otherwise, the t_n_ value is indicated in the column “Comments”.

**Table 3:** Poisson’s ratio results for non-carbon nanotubes reported in the literature.

Approach	Year	Reference	Method	Type of NTs ^1^		ν	Comment
Atomistic	1998	Hernandez et al. [60]	TBMD	BN	(n, n)	0.260	average value
					(n, 0)	0.240	
	2004	Jeng et al. [38]	MD: TB many body potential	GaN	(5, 5)	0.263	–
					(9, 0)	0.221	
	2007	Verma et al. [55]	MD: TB potential	BN	(n, n), (n, 0)	0.140	average value
	2017	Kochaev [37]	ab initio	BN	(10, 10)	0.560	–
					(10, 0)	0.570	
				AlN	(10, 10)	0.520	
					(10, 0)	0.550	
				GaN	(10, 10)	0.530	
					(10, 0)	0.550	
				AlP	(10, 10)	0.510	
					(10, 0)	0.510	
				GaP	(10, 10)	0.510	
					(10, 0)	0.520	
CM	2010	Oh [62]	CL thermodynamic approach + TB potential	BN	(n, n)	0.150	converged average value
					(n, 0)	0.170	
NCM/MSM	2015	Ansari et al. [66]	analytical solution	BN	(n, n), (n, 0)	0.217	average value
	2016	Jiang and Guo [39]	“stick-and-spring” model + analytical	BN	(n, n)	0.216	converged average value
					(n, 0)	0.219	
				AlN	(n, n)	0.281	
					(n, 0)	0.287	
				GaN	(n, n)	0.285	
					(n, 0)	0.290	
				BP	(n, n)	0.360	
					(n, 0)	0.365	
				GaP	(n, n)	0.428	
					(n, 0)	0.435	
				InP	(n, n)	0.455	
					(n, 0)	0.460	
				SiC	(n, n)	0.095	
					(n, 0)	0.100	
	2019	Genoese et al. [41]	“stick-and-spring” model + Donnell thin shell model	BN	(n, n)	0.239	converged average value
					(n, 0)	0.226	
				SiC	(n, n)	0.330	
					(n, 0)	0.331	
	2021	Sakharova et al. [36]	beams + FE model	BN	(n, n); (n, 0); (n, m)	0.150	converged average value
Experimental	2005	Hung et al. [16]	NS + analytical	SWGaNNTs		0.242	–

^1^ All theoretical results presented were obtained for single-walled NTs.

**Table 4:** Vibrational properties of N-CNTs available in the literature.

Approach	Year	Reference	Method	Type of NTs		Support Case	L_n_, nm	*f*_n1_, THz	Comments
Atomistic	2015	Ansari and Ajori [57]	MD: TB potential	BN	(10, 10)	CF	6	0.145	The BNNTs with clamped-clamped support have higher values of $f_{n1}$ than those with cantilevered support. The first fundamental frequency decreases for small nanotube length and then tends to stabilize for NT length L_n_ > 4 nm.
							8	0.081	
							10	0.048	
							12	0.016	
						CC	6	0.855	
							8	0.532	
							10	0.274	
							12	0.210	
	2015	Chandra et al. [48]	MD: Tersoff-type potential	BN	(10, 10)	CC	7	0.52	The values of *f*_n1_ were determined at T = 400K. The bigger the BNNT length, the higher the first fundamental frequency.
							21	0.06	
CM	2013	Panchal et al. [63]	thin wall tube (outer diameter of 0.8 nm, thickness of 0.065 nm) + analytical	BN	–	CF	6	0.744	The values of *f*_n1_ were obtained for the case of attached mass at free NT end of 10^−8^ fg (from the range of 10^−8^ to 10^−2^ fg). The *f*_n1_ value increases with decreasing of the attached mass and NT length.
							8	0.419	
							10	0.268	
NCM/MSM	2006	Li and Chou [34]	beams + FE model	BN	(4, 4)	CF	7	0.044	The first fundamental frequency values decrease with increased in the nanotube length and diameter. The decrease rate is higher in the case of clamped-clamped support.
							9	0.030	
							11	0.019	
							13	0.015	
						CC	7	0.289	
							9	0.178	
							11	0.122	
							13	0.089	
					(7, 0)	CF	7	0.044	
							9	0.030	
							11	0.019	
							13	0.015	
						CC	7	0.281	
							9	0.174	
							11	0.119	
							13	0.085	
	2010	Chowdhury et al. [98]	unspecified elastic elements	BN	(4, 4)	CC	6	1.647	The *f*_n1_ frequency decreases with increasing NT length, L_n_, and diameter, D_n_.
							8	1.253	
							9	1.118	
							10	0.941	
					(8, 0)		6	1.794	
							8	1.382	
							10	1.029	
							12	0.824	
	2013	Panchal et al. [67]	beams + FE model	BN	(5, 5)	CF	6	0.077	The values of *f*_n1_ de-crease with increasing L_n_ and D_n_ The fundamental frequencies for high-er-order vibrational modes were also cal-culated.
							9	0.037	
							12	0.021	
							13	0.016	
						CC	6	0.426	
							9	0.227	
							12	0.135	
							13	0.107	
	2014	Khani et al. [100]	beams + FE model	SiC	(15, 15)	CF	4	0.025	The *f*_n1_ frequency decreases with increasing SiCNT length.First five vibrational modes shapes were analysed and respective values of the fundamental frequencies were calculated. The fundamental frequencies are higher for the case of clamped-clamped support.
							6	0.021	
							8	0.011	
							10	0.008	
						CC	4	0.062	
							6	0.044	
							8	0.029	
							10	0.025	
					(13, 0)	CF	4	0.024	
							6	0.013	
							8	0.006	
							10	0.004	
						CC	4	0.060	
							6	0.039	
							8	0.023	
							10	0.015	
	2016	Giannopoulos et al. [69]	springs + FE model	BN	(12, 12)	CF	8	0.210	The values of *f*_n1_ decrease with increasing NT length, L_n_, and diameter, D_n_. The fundamental frequencies for first three modes were calculated. The *f*_n_ values decrease with increasing of the vibrational mode order. The fundamental frequencies are higher for the case of clamped-clamped support.
							16	0.124	
							25	0.073	
							35	0.061	
						CC	8	0.408	
							16	0.256	
							25	0.171	
							35	0.123	
					(20, 0)	CF	8	0.207	
							16	0.125	
							24	0.081	
							32	0.064	
						CC	8	0.467	
							16	0.256	
							24	0.174	
							32	0.127	
	2017	Ansari and Rouhi [101]	beams + FE model	SiC	(5, 5)	CF	4	0.359	The first fundamental frequency, *f*_n1_ decreases for small values of the NT lengths and then tends to stabilize for L_n_ > 3 nm. The values of *f*_n1_ obtained for clamped-clamped support are about two times higher than those obtained for clamped-free support.
							6	0.236	
							8	0.154	
							9	0.103	
						CC	4	0.780	
							6	0.565	
							8	0.458	
							9	0.390	
					(9, 0)	CF	4	0.462	
							6	0.205	
							8	0.134	
							9	0.103	
						CC	4	0.878	
							6	0.565	
							8	0.390	
							9	0.390	
	2019	Yan et al. [71]	analytical solution + Euler beam theory	BN	(5, 5)	CF	5	1.052	The values of *f*_n1_ decrease with increasing L_n_. *f*_n1_ slightly increases for small NT diameters and then tends to nearly constant value for D_n_ > 1.0 nm.
							6	0.918	
							7	0.731	
						CC	5	2.127	
							6	1.860	
							7	1.487	
	2022	Zakaria [68]	two-section beams + FE model	BN	(12, 12)	SS	8	0.189	The values of *f*_n1_ decrease with increasing NT length, D_n_, and diameter, D_n_.
							16	0.054	
							25	0.022	
							35	0.012	
					(20, 0)		8	0.179	
							16	0.048	
							24	0.023	
							32	0.012	

## Data Availability

The data presented in this study are available on request from the corresponding author after obtaining permission of authorized person.
